# Advection Drives Nitrate Past the Microphytobenthos in Intertidal Sands, Fueling Deeper Denitrification

**DOI:** 10.3389/fmicb.2021.556268

**Published:** 2021-06-17

**Authors:** Charles A. Schutte, Paulina Huanca-Valenzuela, Gaute Lavik, Hannah K. Marchant, Dirk de Beer

**Affiliations:** ^1^Microsensors Group, Max Planck Institute for Marine Microbiology, Bremen, Germany; ^2^Biogeochemistry Department, Max Planck Institute for Marine Microbiology, Bremen, Germany

**Keywords:** denitrification, microphytobenthos, sand flat, nitrate uptake, permeable sediment

## Abstract

Nitrification rates are low in permeable intertidal sand flats such that the water column is the primary source of nitrate to the sediment. During tidal inundation, nitrate is supplied to the pore space by advection rather than diffusion, relieving the microorganisms that reside in the sand from nitrate limitation and supporting higher denitrification rates than those observed under diffusive transport. Sand flats are also home to an abundant community of benthic photosynthetic microorganisms, the microphytobenthos (MPB). Diatoms are an important component of the MPB that can take up and store high concentrations of nitrate within their cells, giving them the potential to alter nitrate availability in the surrounding porewater. We tested whether nitrate uptake by the MPB near the sediment surface decreases its availability to denitrifiers along deeper porewater flow paths. In laboratory experiments, we used NO_*x*_ (nitrate + nitrite) microbiosensors to confirm that, in the spring, net NO_*x*_ consumption in the zone of MPB photosynthetic activity was stimulated by light. The maximum potential denitrification rate, measured at high spatial resolution using microsensors with acetylene and nitrate added, occurred below 1.4 cm, much deeper than light-induced NO_*x*_ uptake (0.13 cm). Therefore, the shallower MPB had the potential to decrease NO_*x*_ supply to the deeper sediments and limit denitrification. However, when applying a realistic downward advective flow to sediment from our study site, NO_*x*_ always reached the depths of maximum denitrification potential, regardless of light availability or season. We conclude that during tidal inundation porewater advection overwhelms any influence of shallow NO_*x*_ uptake by the MPB and drives water column NO_*x*_ to the depths of maximum denitrification potential.

## Introduction

Coastal ecosystems receive massive inputs of nitrogen from land that can fuel algal primary production and cause harmful algal blooms and hypoxia (Rabalais, 2002; Gruber and Galloway, 2008). Denitrification removes bioavailable nitrogen from the biosphere, returning it to the atmosphere as inert nitrogen gas (N_2_). Denitrification rates are usually limited by the rate of nitrate supply by diffusion or advection (Aller, 1982; Joye and Anderson, 2008). In permeable coastal sediments, advection is driven by a combination of faunal bioirrigation and current-driven pressure gradients over ripples, transporting oxygen and nitrate centimeters deep into the sand (Huettel et al., 1996; Billerbeck et al., 2006; Werner et al., 2006; Ahmerkamp et al., 2017). As a result, advection can increase areal denitrification rates by an order of magnitude compared with diffusive transport in the same sediment (Gao et al., 2012). However, not only denitrifying microorganisms consume nitrate in these sediments. The microphytobenthos (MPB) also competes for available nitrate (Lomstein et al., 1990; Sundbäck et al., 1991; Lorenzen et al., 1998; Longphuirt et al., 2009; Stief et al., 2013). Diatoms are often an important component of the MPB (Longphuirt et al., 2009; Stief et al., 2013). They are known to take up and store nitrate at high concentrations within their cells (Lomas and Glibert, 2000; Kamp et al., 2011, 2015; Merz et al., 2020). The influence of the MPB on denitrification in coastal sediment has been investigated in the past under conditions where mass transport was driven by diffusion rather than advection (Nielsen et al., 1990; Risgaard-Petersen et al., 1994; Lorenzen et al., 1998; Sundbäck and Miles, 2000; Risgaard-Petersen, 2003). In general, algal ammonium uptake led to nitrogen limitation of nitrification, which in turn provided less nitrate substrate for denitrification, thereby slowing rates of coupled nitrification-denitrification (Risgaard-Petersen, 2003).

We studied how nitrate uptake by the MPB affects denitrification in an intertidal sand flat with permeable sediments and porewater advection during tidal inundation. Here, low nitrification rates limit the importance of coupled nitrification-denitrification (Marchant et al., 2014), hence denitrification is controlled by the nitrate supply from the water column. While the nitrate supply is ample in the winter when water column nitrate concentrations are typically in excess of 50 μmol L^–1^, it is possible that nitrate is limiting in the summer when water column concentrations are 1–2 μmol L^–1^ or less (Grunwald et al., 2010). Therefore, denitrification is most likely limited by nitrate availability in the summer. The decrease from winter maximum to summer minimum water column nitrate concentrations is driven by spring phytoplankton blooms, some of which are deposited on shallow sediments (Lomstein et al., 1990). This corresponds with the end of the time period, between fall and spring, when phytopigment and intracellular nitrate concentrations are highest in intertidal sand flats (Stief et al., 2013). Therefore, intense springtime nitrate uptake by the MPB may also decrease porewater nitrate concentrations. In summary, it is most likely that MPB nitrate uptake can limit the amount of nitrate available for denitrification during the spring and summer.

We tested the hypothesis that, at high tide when water column nitrate is driven into the sediment by advection, the MPB intercepts nitrate near the sediment surface and decreases its availability for denitrifying microorganisms located deeper into the sand. In laboratory experiments, we used NO_*x*_ (nitrate + nitrite) and oxygen microsensor measurements to test our hypothesis. We measured the depths in the sediment at which net oxygen production in the light and potential denitrification rates were highest to show a vertical separation between the two processes. We then measured oxygen and NO_*x*_ penetration depths in the light and the dark across a range of imposed nitrate fluxes into the sediment to directly demonstrate whether nitrate uptake activity at the sediment surface was sufficient to alter NO_*x*_ availability deeper in the sediment.

## Materials and Methods

### Field Sampling

The Janssand intertidal sand flat is located in the German Wadden Sea, between the mainland and Spiekeroog Island. The site is characterized by fine quartz sand (mean grain size 176 μm, high permeabilities of 7.2–9.5 × 10^–12^ m^2^, and low sediment total organic carbon content between 0.04 and 0.11% (Billerbeck et al., 2006), and a porosity of 0.38 (Gao et al., 2012). All samples were collected from the upper portion of the flat near its eastern edge (53°44.236′N, 7°41.818′E). With a typical tidal range of around 3 m, during high tide the upper flat is inundated by 1.5–2 m of water while during low tide it is exposed for 6–8 h (Billerbeck et al., 2006). Sediment cores were collected from areas with dense brown diatom mats. On a preliminary sampling trip in March 2016, a 1 cm^3^ sediment subsample was collected from the top 2 cm of each core and mixed with 5 mL of fixative (2% formaldehyde, 1% potassium iodine in nitrate-free artificial seawater (ASW)) and stored at 4°C for diatom cell counting in 6 mL exetainers. The samples were mixed by inversion, allowed to settle, and the supernatant was decanted. The sediment was rinsed in this manner three more times with 2 mL of ASW, and the supernatant was combined. The number of diatoms in ten 1 μL volumes was counted using an inverted microscope at 320x magnification (Lund et al., 1958). The mean diatom abundance was 28 ± 7 × 10^4^ cells cm^–3^ (*n* = 22), an order of magnitude higher than previously measured at this site (Marchant et al., 2014), indicating that it was possible to visually target areas with high diatom abundance for sampling.

The sediment cores used in the following measurements were collected during tidal flat exposure in April and August of 2016 and February of 2017, when water temperatures were 9, 21, and 3°C, respectively. Around 15 cm of sediment was collected in clear, 8.3 cm diameter and 30 cm tall core tubes. In each season, 3 sediment cores were collected haphazardly from within a ∼0.5 m^2^ area with visible microphytobenthos and no visible macrofauna or bioturbation activity. NO_*x*_ concentrations (ΣNO_2_^–^, NO_3_) were determined using a chemiluminescence detector after reduction to NO with 90°C acidic Vanadium (III) chloride (Braman and Hendrix, 1989). *In situ* NO_*x*_ concentrations were 15, 1.5, and 10 μmol L^–1^, in April 2016, August 2016, and February 2017, respectively. Sediment cores were immediately transported in a dark cooler to the laboratory. They were stored at *in situ* temperatures under an automated daily light/dark illumination cycle that was programmed to match the time of year at the field site, with a light intensity of 100 μmol photons m^–2^ s^–1^. This light intensity was close to *in situ* values during inundation (Billerbeck et al., 2007). Seawater collected from the study site was percolated down through each core twice per day (every 12 h, ∼8 a.m. and 8 p.m.) by opening a valve at the bottom of the core and gravity-draining the water. This replaced the stagnant porewater in the top 5 cm of the sediment, simulating natural, tidal patterns of porewater flushing that regularly expose the microbial community to oxygen and nitrate (Werner et al., 2006). Around 0.5 cm of seawater was maintained above the sediment surface at all times to prevent the core from drying out. Seawater NO_*x*_ concentrations were monitored and kept at *in situ* values for all experiments. Experiments were completed between 1 day and 2 weeks after sample collection. This time period was chosen to balance the need for replication of time-consuming microsensor measurements with potential changes in the sediment microbial community. The careful control of laboratory conditions described above with respect to temperature, light/dark cycles, and porewater flushing were implemented to minimize these changes, but the biomass and composition of the microbial community were not tracked during these experiments. The use of clear core tubes may have allowed algal growth on the internal core tube walls, though no visible growth was observed in any sediment core. Moreover, all microsensor measurements were made in the center of these 8.3 cm diameter core tubes, at least 3 cm from the nearest tube wall, minimizing any influence on porewater chemistry at the measurement location.

All measurements made using these sediment cores, described in detail below, were completed during the daylight phase of the artificial day/night cycle, typically beginning 2–4 h after dawn. Because experimental manipulations involved percolating surface seawater through the core, they took the place of the regular, morning (8 am) percolation event meant to simulate tidal inundation. Therefore, measurements typically began 12–14 h following the previous (8 pm) percolation event. Because simulated tidal inundation in the laboratory was carried out by hand, it was not synchronized with the timing of tidal inundation at the field site as this would have required large shifts in the relative timing of light and inundation over a 2-week measurement period.

### Microsensor Measurements of Volumetric NO_*x*_ Consumption Rates

Oxygen and NO_*x*_ consumption rates were measured using oxygen microsensors (Revsbech, 1989) and NO_*x*_ microbiosensors as described previously (Larsen et al., 1996), using a microsensor set up with a motorized micromanipulator and computerized data acquisition. Initially, each sediment core was placed in the light (∼100 μmol photons m^–2^ s^–1^). The sediment surface with respect to the sensor tips was determined using a dissecting microscope. Two consecutive oxygen and NO_*x*_ microprofiles were then measured ∼20 min apart to confirm that the profiles were at steady state. At 0.1–0.2 cm depth an oxygen maximum was observed in these steady state microprofiles, indicating the maximum photosynthetic activity.

The microsensors were then positioned at the depth of the oxygen maximum. Oxygenated overlying seawater containing ambient levels of NO_*x*_ was rapidly gravity-drained through the core, until the oxygen and NO_*x*_ concentrations at the target depth matched those in the overlying water. Water flow was then turned off, and volumetric NO_*x*_ and oxygen production and consumption rates were obtained from linear regression of concentration versus time during the 10 min measurement window. All regressions were significant (*p* < 0.01) with *r*^2^-values greater than 0.95. These measurements were made on two independent cores each in the spring and summer.

### Depth Profiles of Potential Denitrification Rates

Depth profiles of potential denitrification rates in the sediment were measured using a modified version of the acetylene block technique (Sørensen, 1978) in one core each in the summer and the winter. Acetylene blocks the last step in denitrification, the reduction of nitrous oxide (N_2_O) to N_2_, allowing N_2_O to accumulate (Yoshinari and Knowles, 1976). This technique was combined with the method for measuring oxygen consumption rate profiles in permeable sediment (Polerecky et al., 2005) by adding a N_2_O microsensor (Andersen et al., 2001). A volume of 160 mL of oxygenated site seawater, amended with nitrate to 20 μmol L^–1^, was added to the core above the sediment. The overlying water was gravity-drained down through the core in 3–4 min. Subsequently, repeated oxygen and N_2_O profiles from 0 to 4 cm depth at 0.1 cm resolution were measured approximately every 18 min. This process was repeated on the same core, this time with 20% (32 mL) of the overlying seawater volume (160 mL) replaced with acetylene-saturated seawater. Complete inhibition of N_2_O reduction has been observed at an acetylene concentration of 0.1 atmospheres (Yoshinari and Knowles, 1976), which can be achieved by replacing 10% of a given seawater volume with acetylene-saturated seawater (Seitzinger et al., 1993). Repeated oxygen and N_2_O microprofiles were measured as described above until consecutive microprofiles were nearly identical (around 2.5 h in the summer and 10 h in the winter).

Denitrification rates were calculated from the N_2_O profiles by solving Equation 1 for R

(1) dC/dt=R-J+o⁢u⁢tJi⁢n

where dC/dt is the change in N_2_O concentration with respect to time, R is the denitrification rate, and J_*out*_ and J_*in*_ are the diffusive fluxes of N_2_O out of and into the volume of sediment centered on the point where the concentration measurement was made. dC/dt at each depth was calculated as the slope of the linear regression of N_2_O concentration versus time. A minimum of 3 N_2_O measurements was used to calculate dC/dt. The diffusive fluxes were calculated using Equation 2 as described by Deurer et al. (2008).

(2) J=-φD4/3(dC/dz)W

where φ is the dimensionless sediment porosity (0.38), Dw is the diffusion coefficient for N_2_O in water at 20°C, and dC/dz is the N_2_O concentration gradient with respect to depth. We assumed a diffusion coefficient of 6.58 × 10^–6^ m^2^ h^–1^ (Tamimi et al., 1994). dC/dz was estimated as the difference in concentration between the depth where the calculation was made and the depth above it (J_*out*_) or below it (J_*in*_) divided by the change in depth (0.001 m). Since multiple N_2_O profiles were measured during the time that dC/dt was constant, the net N_2_O flux out of each depth (J_*out*_ – J_*in*_) was calculated for each profile and the mean flux was used to calculate denitrification rates. Denitrification rates are expressed on a per mole of nitrogen (not N_2_O) basis.

### Potential Denitrification Rate Kinetics

This modified acetylene block technique was also used to estimate denitrification reaction kinetics. To do this, denitrification rates were calculated at each depth and time point where an N_2_O concentration measurement was made. This rate was estimated at each depth as the first derivative of N_2_O concentration versus time. The first derivative at each time was approximated as the slope of the curve’s tangent line at that point following Equation 3 below (Wood, 1982).

(3) dC/dt=n[NO2-(n+1)NO2](n-1)/[t-(n+1)t](n-1)

At any given depth in the profile, dC/dt_*n*_ is the change in N_2_O concentration with time in profile n, N_2_O_(n__+__1__)_ is the N_2_O concentration measured in the next profile (profile n + 1), N_2_O_(n__–__)_ is the N_2_O concentration measured in the preceding profile (profile n−1), t_(__*n*__+__1__)_ is the time at which the N_2_O measurement was made in profile n + 1, and t_(__*n*__–__1__)_ is the time at which the N_2_O measurement was made in profile n−1. Denitrification rates were calculated from dC/dt_*n*_ using Equation 1. These denitrification rates are calculated for a single point in time. The rates measured in this section are referred to as instantaneous denitrification rates throughout the manuscript to differentiate them from the rates calculated in section “Depth Profiles of Potential Denitrification Rates.”

The apparent porewater NO_*x*_ concentration was estimated at each depth and time point for comparison with the instantaneous denitrification rates. These concentrations were calculated based on the total amount of N_2_O produced and the original 20 μmL L^–1^ pool of NO_*x*_ available in the porewater according to Equation 4.

(4) NO=x(a⁢p⁢p)NO-x(i⁢n⁢i⁢t)2*NO*2f

Where NO_*x*__(app)_ is the apparent NO_*x*_ concentration remaining in the porewater at a given time and depth, NO_*x*__(init)_ is the initial porewater NO_*x*_ concentration (20 μmol L^–1^) added at the start of the incubation, N_2_O is the measured N_2_O concentration (multiplied by 2 to account for denitrification reaction stoichiometry), and f is a correction factor that accounts for the fraction of NO_*x*_ that was consumed by processes other than denitrification (calculated according to Equation 5 below).

(5) f=1+(1-2*NO2/m⁢a⁢xNO)x(init)

Where N_2_O_*max*_ is the maximum N_2_O concentration measured at a particular depth during any time point. This calculation assumes that all or nearly all the initial NO_*x*_ available was consumed by some combination of processes over the course of the ∼2.5-h incubation.

Finally, denitrification reaction kinetics were calculated by fitting the instantaneous denitrification rate versus apparent porewater NO_*x*_ concentration data using the Hill equation (Hill, 1913) (Equation 6 below).

(6) R=(V[NO]x⁢(a⁢p⁢p)m⁢a⁢x*)n/([K]M+n[NO]x⁢(a⁢p⁢p))n

Where R is the instantaneous denitrification rate derived from Equations 3 and 1 and NO_*x(app)*_ is the apparent NO_*x*_ concentration derived from Equation 4. V_*max*_ is the maximum possible rate, K_*M*_ is the half saturation constant, and n is the Hill coefficient. The Hill coefficient is a representation of the relative degree of interaction, or cooperativity, between multiple substrate molecules binding with an enzyme (Monod et al., 1965). The Hill equation has been used to describe the kinetics of allosteric enzymes (Weiss, 1997), and has recently been adapted to model microbial growth rates under substrate-limiting conditions (Arranz and Peinado, 2017). The Hill equation was used instead of the simpler Michaelis-Menten equation (Michaelis and Menten, 1913, 2013) because it yielded a better fit to our data (Akaike information criterion (AIC) values of 39.2 and 90.5, respectively).

### Influence of the MPB on the Depth of NO_*x*_ Penetration Into the Sediment

The MPB microbial community is heterogeneously yet broadly distributed across the intertidal sand flats at this study site (Chennu et al., 2013), making it impossible to collect control sediment cores that would be devoid of MPB activity. Instead, we opted to focus on sediments with the highest MPB density, identified as described in section “Field Sampling.” Our experimental approach centered on creating conditions that maximized the potential of the MPB to alter NO_*x*_ availability at the depth of maximum denitrification activity. If NO_*x*_ penetrated to that depth under these idealized conditions, then we consider it very unlikely that the MPB had any substantial influence on substrate availability for denitrification under either laboratory or *in situ* conditions.

NO_*x*_ and oxygen penetration depths were measured using oxygen microsensors and NO_*x*_ microbiosensors. An initial microprofile was measured with no porewater flow. Then overlying seawater containing oxygen and ambient NO_3_ was pumped down through the core, at either low or high flow rates. Low flow was defined as a porewater infiltration rate of 0.11 mL min^–1^ in all experiments, high flow was 0.36 mL min^–1^ in the spring and 1.3 mL min^–1^ in the summer. These porewater flow rates were equivalent to vertical seawater infiltration rates of 1.2, 4.0, and 14.4 L m^–2^ h^–1^, respectively. The imposed NO_*x*_ flux into the sediment was 18–60 μmol m^–2^ h^–1^ in the spring and 2–22 μmol m^–2^ h^–1^ in the summer. All measurements were done in both the dark and at a light intensity of 100 μmol photons m^–2^ s^–1^, to test the influence of MPB nitrate uptake on nitrate penetration.

Continuous microprofiling began immediately after the onset of downward porewater flow and continued for 3–6 h with consecutive microprofiles measured at 30–60-min intervals. For each microprofile measured, the NO_*x*_ and oxygen penetration depths were calculated as the first depth at which the concentration reached zero. The penetration depth appeared to gradually increase with ongoing percolation. Therefore, the penetration depth was plotted against the elapsed time since the onset of porewater flow and fit with an asymptotic curve of the form:

(7) d=a-b(c)t

where *d* is the penetration depth, *t* is the time elapsed since the start of pumping, and *b* and *c* are dimensionless coefficients used to fit the curve, and *a* is the estimate of the asymptote. This asymptote is the maximum penetration depth, estimated using a least squares fit of Equation 7 (calculated using the non-linear least squares function in R) to the experimentally derived penetration depth versus time curves.

## Results

### NO_*x*_ Consumption Rates by MPB

**FIGURE 1:**
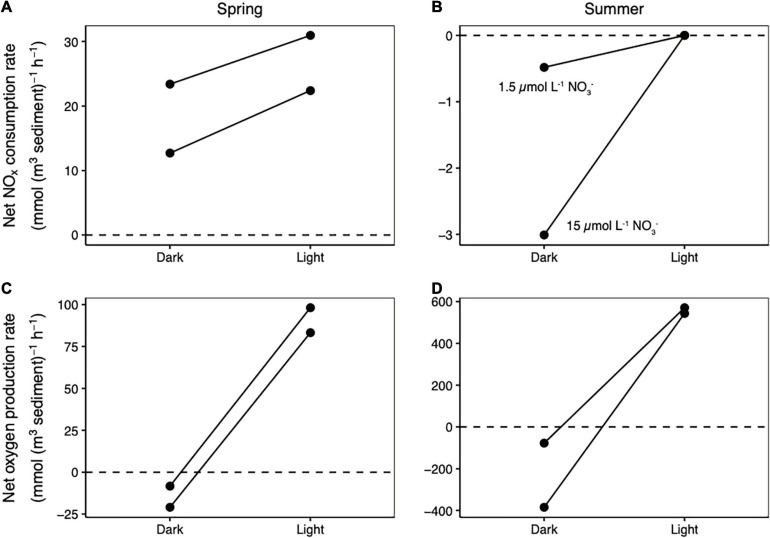
Changes in net NO_*x*_ consumption rates between dark and light conditions measured in the spring **(A)** and summer **(B)**. Changes in net oxygen production rates between dark and light conditions measured in the spring **(C)** and summer **(D)**. A line connects the dark and light measurements made on each core. Negative net consumption rates in **(B)** indicate net NO_*x*_ production.

In the zone of highest photosynthetic activity, NO_*x*_ consumption rates were higher in the light than in the dark, but only in the spring (Figure 1A). In spring, a switch from the dark to the light increased net NO_*x*_ consumption rates from 13 mmol N (m^3^ sediment) ^–1^ h^–1^ to 28 mmol N (m^3^ sediment) ^–1^ h^–1^ in one core (a 1.8-fold increase) and from 23 mmol N (m^3^ sediment) ^–1^ h^–1^ to 31 mmol N (m^3^ sediment) ^–1^ h^–1^ in a duplicate core (a 1.3-fold increase). In contrast, in summer, net NO_*x*_ consumption was not observed in either the light or the dark (Figure 1B), even when nitrate levels were increased to far above ambient summer concentrations (from 1.5 to 20 μmol L^–1^). Net oxygen production and consumption rates were an order of magnitude higher in the summer than the spring (Figures 1C,D).

### Depth Separation of Photosynthesis and Potential Denitrification

**FIGURE 2:**
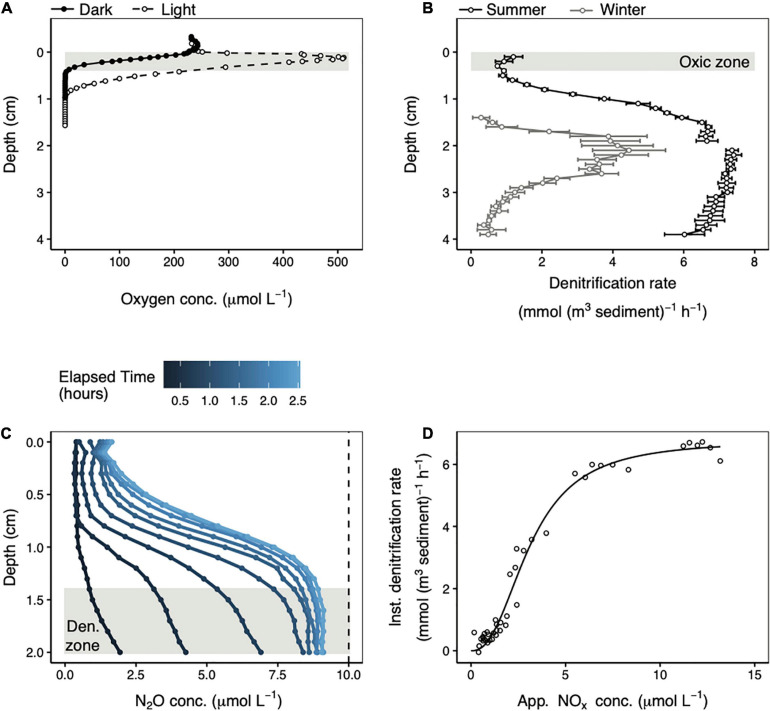
Representative summer steady state oxygen concentration depth profiles in the light (open circles) and in the dark (closed circles) **(A)**. Potential denitrification rate profiles in the summer (black) and in the winter (gray) measured in the light **(B)**. Error bars represent the linear regression standard error. The gap in the summer profile reflects two separate measurements made over different depth intervals in the same core. The shaded areas in **(A,B)** represent the depth interval over which oxygen was always present in steady state profiles. Repeated N_2_O concentration microprofiles following acetylene addition in the summer **(C)**. The dashed line represents the maximum possible N_2_O concentration that could be generated from the available porewater NO_*x*_. The instantaneous potential denitrification rate plotted against the apparent amount of NO_*x*_ remaining in the porewater **(D)** over the 1.4–2.0 cm depth range highlighted in **(C)**. The black line is fit to the data using Equation 6.

In the summer, maximum denitrification occurred far below the zone where the MPB were photosynthetically active (Figure 2). Whereas the photosynthetic oxygen maximum was found at 0.13 cm depth, denitrification peaked below 1.4 cm. Denitrification also occurred within the depth interval that was always exposed to oxygen (0–0.4 cm), but at a much lower level. The mean (± standard deviation) denitrification rate across 0–0.4 cm depth was 0.95 ± 0.19 mmol N (m^3^ sediment) ^–1^ h^–1^. The potential denitrification rate increased steadily with depth down to about 1.4 cm after which it stabilized at values from 5.9 to 7.4 mmol N (m^3^ sediment) ^–1^ h^–1^. The mean denitrification rate over this depth range (1.4–3.9 cm) was 6.9 ± 0.4 mmol N (m^3^ sediment)^–1^ h^–1^. In the winter the potential denitrification rates were lower, particularly deeper in the core. However, the pattern of low potential denitrification rates near the sediment surface which increased to a maximum by 1.5–2 cm depth was identical in both seasons (Figure 2B).

### Potential Denitrification Reaction Kinetics

Potential denitrification reaction kinetics were calculated from the same denitrification rate profiles (measured in summer in the light) described in section “Depth Separation of Photosynthesis and Potential Denitrification.” Focusing on a depth interval with stable, high denitrification rates (1.4–2.0 cm), it is apparent that when acetylene blocked the last step in denitrification, N_2_O accumulated rapidly during the first hour of incubation and slowly approached a maximum value thereafter (Figure 2C). The maximum amount of N_2_O that could be produced was 10 μmol L^–1^ based on the 20 μmol L^–1^ NO_*x*_ added to the seawater medium. The mean N_2_O concentration across these depths at the last time point measured was 9.1 ± 0.1 μmol L^–1^, or around 91% of the maximum possible. Therefore, at the depth of maximum potential denitrification rates, more than 90% of the available NO_*x*_ was consumed by denitrification.

Instantaneous potential denitrification rates increased non-linearly as a function of apparent porewater NO_*x*_ concentrations (Figure 2C). These rates are called “instantaneous” to distinguish them from the potential denitrification rates from the preceding paragraph that were calculated as the change in N_2_O concentration over time at each depth. Instead, instantaneous rates were estimated at a given point in time from the derivatives of individual N_2_O concentration profiles. The non-linear increase in instantaneous potential denitrification rates as a function of substrate concentration was modeled using the Hill equation (Equation 6). Fitting this curve to the data yielded a half-saturation constant (K_*M*_) of 3.1 μmol L^–1^, a maximum instantaneous denitrification rate (V_*m*__*ax*_) of 6.8 mmol N (m^3^ sediment) ^–1^ h^–1^, and a Hill coefficient of 2.4. The upper inflection in the curve in Figure 2C occurs at a rate of around 6.1 mmol N (m^3^ sediment) ^–1^ h^–1^, which is 90% of V_*max*_, and corresponds with an apparent NO_*x*_ concentration of ∼7.8 μmol L^–1^. Above this NO_*x*_ concentration, the instantaneous denitrification rate increased very slowly as a function of increasing NO_*x*_ concentration.

### Influence of the MPB on the Depth of NO_*x*_ Penetration Into the Sediment

**FIGURE 3:**
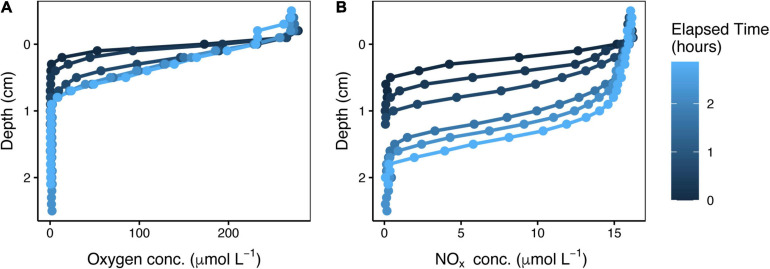
Time series of oxygen **(A)** and NO_*x*_ **(B)** depth profiles after onset of downward water flow through a sediment core. These representative profiles are from the spring, in the dark, with a low flow rate of 0.11 mL min^–1^.

When overlying seawater containing oxygen and NO_*x*_ was pumped down through a sediment core at a constant rate, these electron acceptors penetrated gradually deeper but their concentration gradients consistently remained steep (see representative profiles in Figure 3). There was ample NO_*x*_ available in the porewater just a few millimeters above the NO_*x*_ penetration depth regardless of the amount of time elapsed since the onset of pumping. Therefore, the NO_*x*_ penetration depth serves as a proxy for the depth range over which denitrification was possible.

**FIGURE 4:**
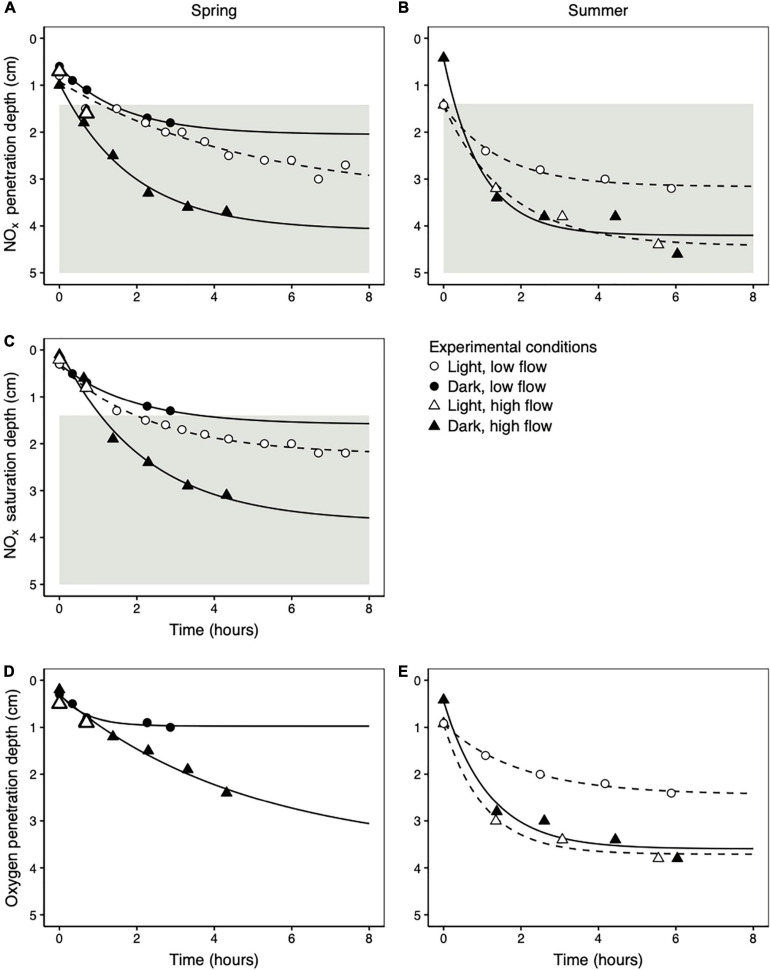
The change in NO_*x*_ penetration depths **(A,B)**, NO_*x*_ saturation depths (the depth at which the NO_*x*_ concentration falls below 7.8 μmol L^–1^) **(C)**, and oxygen penetration depths **(D,E)** as a function of time following the onset of porewater flow. Data from the spring are displayed in the left column **(A,C,D)** and data from the summer are displayed in the right column **(B,E)**. All summer profiles were measured in the same core. In the spring, low flow rate profiles were measured in one core and high flow rate profiles in a second, independent core. The lines represent asymptotic fits to the data according to Equation 7. The gray areas in **(A–C)** represent the deep depth interval over which potential denitrification rates reached their maximum (below 1.4 cm; Figure 2B).

NO_*x*_ penetration depths in the light and dark were directly compared in three separate cores, once at low flow in the spring, once at high flow in the spring, and once at high flow in the summer. In all cases there was no difference in NO_*x*_ penetration between the light and dark treatments (Figures 4A,B). However, flow rate did have an effect; NO_*x*_ penetration depths were much higher at high flow rates than at low flow rates in both the spring (Figure 4A; different flow rates measured in independent cores) and the summer (Figure 4B; measured in the same core). Similarly, flow rate had a much stronger influence on the NO_*x*_ saturation depth than light (Figure 4C). The NO_*x*_ saturation depth is the depth at which the porewater NO_*x*_ concentration fell below the saturating concentration of 7.8 μmol L^–1^ required to drive potential denitrification rates at 90% of V_*max*_ (see section “Potential Denitrification Reaction Kinetics” for details). Under all conditions tested in the spring, saturating NO_*x*_ concentrations reached the depth of maximum denitrification potential (Figure 4C). This deep NO_*x*_ penetration occurred within 4 h after the onset of porewater flow.

As with NO_*x*_ penetration depths, oxygen penetration depths were similar at the same flow rate regardless of light availability in both spring (Figure 4D) and summer (Figure 4E). The oxygen penetration depth was consistently shallower than the NO_*x*_ penetration depth during porewater advection (mean ± standard deviation = 0.8 ± 0.4 cm) across all individual profile measurements in all experimental conditions (*n* = 20 profiles after time 0 in Figure 4).

## Discussion

### Light-Induced NO_*x*_ Uptake by the MPB

In the spring, NO_*x*_ was consumed more rapidly in the light than in the dark where the MPB were photosynthetically active (Figure 1A). Stimulation of net NO_*x*_ consumption in the light is consistent with nitrate uptake and storage by diatoms grown in the light (Kamp et al., 2011), and with assimilation into biomass (Middelburg and Nieuwenhuize, 2000). The MPB may further stimulate bacterial nitrogen uptake in the light by exuding organic carbon that can serve as a substrate for heterotrophic activity (Evrard et al., 2008). NO_*x*_ assimilation was more important than dissimilatory respiration in shallow, oxic sediment during the spring. Near the sediment surface, light-induced NO_*x*_ uptake (the difference between net NO_*x*_ uptake measured in the light and the dark) accounted for 29–33% of total net NO_*x*_ consumption. In contrast, denitrification in oxic sediment in any season only accounted for at most 3–4% of total net NO_*x*_ consumption. As DNRA rates at this study site were less than 20% of denitrification rates (Marchant et al., 2014) these were not considered further. The remaining balance of the observed net NO_*x*_ uptake can most likely be attributed to microbial assimilation, by either autotrophs or heterotrophs, in the dark (e.g., Middelburg and Nieuwenhuize, 2000). In summary, dissimilatory NO_*x*_ respiration was less important than MPB-influenced NO_*x*_ uptake near the sediment surface in the spring. No net NO_*x*_ consumption was observed in photosynthetic sediment during the summer in either the light or the dark, even when exposed to an unseasonably high concentration of 20 μmol L^–1^ (Figure 1A). This was despite very high net oxygen production rates (Figure 1B). The most likely explanation for this is that high rates of organic matter remineralization in the summer (Billerbeck et al., 2006) released sufficient ammonium into the porewater to meet the nitrogen demand of the MPB.

### Denitrification Kinetics

Our half-saturation constant (K_*M*_) of 3.1 μmol L^–1^ and maximum instantaneous denitrification rate (V_*max*_) of 6.8 mmol N (m^3^ sediment) ^–1^ h^–1^ fell within the ranges of 1.5–19.8 μmol L^–1^ and 0.9–7.5 mmol N (m^3^ sediment) ^–1^ h^–1^ reported for permeable sediments across six sites in coastal Australia (Evrard et al., 2013). However, we observed a low sensitivity of denitrification rates to increasing NO_*x*_ at low concentrations (Figure 2B) that is not apparent in the data of Evrard et al. (2013). It is possible that at very low porewater NO_*x*_ concentrations, denitrification rates are limited by diffusion-driven mass-transport of NO_*x*_ from the porewater to the surface of sand grains inhabited by microorganisms (Probandt et al., 2018; Ahmerkamp et al., 2020) that are likely the dominant sites of denitrification within the bulk sediment.

### Controls on NO_*x*_ Penetration Depth During Tidal Inundation

Despite high nitrate consumption activity in the top few millimeters of sediment (Figure 1A), when nitrate-bearing seawater was pumped into sediment cores to simulate *in situ* porewater advection (*n* = 7), some NO_*x*_ always penetrated to depths with high denitrification potential regardless of season, light availability, or porewater flow rate (Figures 4A,B and Table 1). NO_*x*_ saturation depths were at most 0.5 cm shallower than NO_*x*_ penetration depths. Moreover, saturating NO_*x*_ concentrations (7.8 μmol L^–1^) penetrated more than 1 cm deep into the sand under all conditions tested. This was the case even though these experiments were designed to maximize the potential influence of the MPB. The range in experimental NO_*x*_ fluxes into the sediment was 2–60 μmol m^–2^ h^–1^, at least an order of magnitude lower than the 700–43,000 μmol N m^–2^ h^–1^ range of nitrate fluxes into the sediment that have been modeled for this site (Gao et al., 2012). Even so, the advective transport of nitrate into the sediment was always higher than nitrate consumption by the MPB such that the MPB could not limit nitrate supply to the denitrifying microbial community.

**TABLE 1:** Theoretical maximum NO_*x*_ and oxygen penetration depths and NO_*x*_ fluxes, under different light and flow conditions.

**Season**	**Light intensity (μmol photons m**^–^**^2^ s**^–^**^1^)**	**Flow (mL min**^–^**^1^)**	**NO_*x*_ Flux (μmol m**^–^**^2^ h**^–^**^1^)**	**NO_*x*_ penetration depth (cm)**	**Oxygen penetration depth (cm)**	**Depth difference (cm)**
Spring	0	0.11	18	2.1 ± 0.1**	1.0 ± 0.1**	1.1 ± 0.1
Spring	100	0.11	18	3.2 ± 0.2**	ND	ND
Spring	0	0.36	60	4.1 ± 0.2**	3.7 ± 1.6	ND
Spring	100	0.36	60	ND	ND	ND
Summer	0	0.11	2	ND	ND	ND
Summer	100	0.11	2	3.1 ± 0.1**	2.4 ± 0.1**	0.7 ± 0.1
Summer	0	1.3	22	4.2 ± 0.3**	3.6 ± 0.2**	0.6 ± 0.1
Summer	100	1.3	22	4.4 ± 0.3*	3.7 ± 0.2*	0.7 ± 0.1

ND indicate no data (due to sensor failures). *Asymptotic fit is significant at the p < 0.05 level. **Asymptotic fit is significant at the p < 0.01 level.

The observation that light-induced MPB activity did not influence the depth of nitrate penetration can be validated by a simple mass balance of nitrate in the zone of benthic photosynthetic activity. This zone extended over a depth range of at most 0.8 cm (the maximum steady state oxygen penetration depth; Figure 2A). Assuming that light induced an increase in the maximum net volumetric NO_*x*_ consumption rate of 8 mmol N (m^3^ sediment) ^–1^ h^–1^ (the difference between light and dark net NO_*x*_ consumption rates in one core in the spring; Figure 2A) in this zone, the maximum increase in the areal net NO_*x*_ consumption rate that can be attributed to the MPB was 64 μmol m^–2^ h^–1^. Even if we apply the maximum net NO_*x*_ consumption rate measured in the spring (31 mmol N (m^3^ sediment) ^–1^ h^–1^) to this entire depth zone, the areal net NO_*x*_ consumption rate comes to 248 μmol m^–2^ h^–1^. This maximum possible rate is only 35% of the low end of the 700–43,000 μmol N m^–2^ h^–1^ range of nitrate fluxes into the sediment that have been modeled for this site (Gao et al., 2012). In summary, we find no evidence that the MPB limited the amount of nitrate available in the sediment for denitrification within our very conservative experimental framework, and we also have no reason to expect that the MPB would limit nitrate availability for denitrification *in situ*.

### Coupling Between NO_*x*_ and Oxygen Penetration Depths

For the porewater flow rates of 0.11, 0.36, and 1.3 mL min^–1^ used in this experiment, water would, in theory, penetrate to 2.6, 8.4, and 30 cm depth, respectively, over the 8 h represented in Figure 4. Measured NO_*x*_ and oxygen penetration depths were much shallower than these values, demonstrating the importance of consumption throughout the sediment in controlling the penetration depth of terminal electron acceptors. During advection, oxygen and NO_*x*_ penetrated easily through shallow, oxidized sand where consumption rates were low, but higher consumption rates in deeper, more reduced sands limited the depth to which they could penetrate. For example, potential denitrification rates were 6–7 times higher in anoxic than oxic conditions (Figure 2A). Therefore, NO_*x*_ penetrated easily through the oxic zone of the sand, but was consumed completely within a few millimeters of the oxygen penetration depth.

Despite differences in penetration depth, oxygen and NO_*x*_ coexisted over a depth range that was typically several centimeters deep during porewater advection (Figure 4). The presence of oxygen does not prevent this NO_*x*_ from being used for denitrification. We measured denitrification rates in the oxic zone similar to those measured in other oxic permeable intertidal and subtidal sediments (Rao et al., 2008; Gao et al., 2010), and denitrifying microorganisms from our study site are capable of simultaneously respiring oxygen and nitrate (Marchant et al., 2017). Moreover, porewater oxygen concentrations ranged between 20 μmol L^–1^ to more than 200 μmol L^–1^ in the oxic zone of our sediment cores in which we detected potential denitrification, similar to the > 100 μmol L^–1^ oxygen background in which denitrification has been observed by others in whole sediment (Marchant et al., 2017) and bacterial cultures (Qu et al., 2016). Therefore, if oxygen and NO_*x*_ profiles overlap substantially during sand flat inundation as they did in our experiments, then denitrification in the oxic zone may play a very important role in the overall nitrogen metabolism of the flat.

## Conclusion

Based on our results, we can reject the hypothesis that MPB covering permeable intertidal sandflats intercepts nitrate near the sediment surface and decreases its availability for denitrifying microorganisms. Though MPB-inhabited sediments do indeed take up nitrate, this uptake is overwhelmed by advective nitrate transport. Therefore, it appears that MPB limitation of denitrification only occurs in sediments where diffusion is the dominant transport process (Lorenzen et al., 1998; Risgaard-Petersen, 2003). Previous studies have found no systematic differences in MPB abundance between diffusive and advective sediments that could account for this difference in the influence of MPB on denitrification (Sundbäck and Miles, 2000; Cartaxana et al., 2006). Our findings emphasize that intertidal permeable sediments are important sites of nitrogen removal via denitrification (Gao et al., 2012; Huettel et al., 2014; Marchant et al., 2014).

## Data Availability Statement

The datasets presented in this study can be found in online repositories. The names of the repository/repositories and accession number(s) can be found below: The data and associated computer code are publicly available as a computing capsule at the Code Ocean repository (doi: 10.24433/CO.1921276.v1).

## Author Contributions

CS led the data analysis and writing efforts. CS and PH-V carried out the experiments. CS, PH-V, and HM analyzed the samples. All authors contributed to the experimental design and to writing and editing the manuscript.

## Conflict of Interest

The authors declare that the research was conducted in the absence of any commercial or financial relationships that could be construed as a potential conflict of interest.
